# Design Strategies of S_8_ Molecule Cathodes for Room-Temperature Na-S Batteries

**DOI:** 10.3390/nano15050330

**Published:** 2025-02-20

**Authors:** Sha-Sha Shi, Zi-Qi Cai, Chen-Kai Lu, Jing Li, Nan-Nan Geng, Dong-Tao Lin, Tao Yang, Tao Liu

**Affiliations:** 1 Guangxi Key Laboratory of Processing for Non-ferrous Metals and Featured Materials, School of Resources, Environment and Materials, Guangxi University, Nanning 530004, China; sss450802@163.com (S.-S.S.); gnn0420@outlook.com (N.-N.G.); 2 Future Technology School, Shenzhen Technology University, Shenzhen 518118, China; 202100501059@stumail.sztu.edu.cn (Z.-Q.C.); luchenkai0818@163.com (C.-K.L.); lijing0708@163.com (J.L.); dongtao_lin@163.com (D.-T.L.); 3 TEMA-Centre for Mechanical Technology and Automation, Department of Mechanical Engineering, University of Aveiro, 3810-193 Aveiro, Portugal

**Keywords:** Na-S batteries, cathode, nanostructure engineering, catalyst strategy, conversion pathway

## Abstract

Sodium–sulfur batteries have been provided as a highly attractive solution for large-scale energy storage, benefiting from their substantial storage capacity, the abundance of raw materials, and cost-effectiveness. Nevertheless, conventional sodium–sulfur batteries have been the subject of critique due to their high operating temperature and costly maintenance. In contrast, room-temperature sodium–sulfur batteries exhibit significant advantages in these regards. The most commonly utilized cathode active material is the S_8_ molecule, whose intricate transformation process plays a crucial role in enhancing battery capacity. However, this process concomitantly generates a substantial quantity of polysulfide intermediates, leading to diminished kinetics and reduced cathode utilization efficiency. The pivotal strategy is the design of catalysts with adsorption and catalytic functionalities, which can be applied to the cathode. Herein, we present a summary of the current research progress in terms of nanostructure engineering, catalyst strategies, and regulating sulfur species conversion pathways from the perspective of high-performance host design strategy. A comprehensive analysis of the catalytic performance is provided from four perspectives: metal catalysts, compound catalysts, atomically dispersed catalysts, and heterojunctions. Finally, we analyze the bottlenecks and challenges, offering some thoughts and suggestions for overcoming these issues.

## 1. Introduction

Alongside the global energy transition, the installation of renewable energy sources continues to increase, placing greater demands on large-scale energy storage systems. In the fundamental composition of large-scale energy storage systems, batteries serve as the core component. Consequently, the selection of battery type exerts a decisive influence on the construction of such power systems. It is imperative to select the most suitable battery type by meticulously evaluating a spectrum of factors, including theoretical capacity, safety, cost, and other pertinent parameters.

Lithium-ion batteries are widely popularized because of their excellent electrochemical energy storage performance. However, due to the limited lithium resources, it is laborious for them to meet the requirements of large-scale energy storage applications. It is urgently needed to explore new energy storage systems that are made of abundant raw materials, cost-effective, and environmentally friendly. Benefiting from advantages in energy density, stability, and longevity, Na-S batteries satisfy the criteria for large-scale application and cost-effectiveness within the context of power grids. Conventional high-temperature Na-S batteries are composed of electrodes in the molten state and an Alumina (β-Al_2_O_3_) electrolyte. However, because β-Al_2_O_3_ exhibits low sodium ionic conductivity at ambient temperature, the operating temperature typically exceeds 300 °C [1].

In accordance with the fundamental reaction 2Na + xS→Na_2_S_x_ (x = 3~5), the ultimate product of the traditional Na-S battery discharge is Na_2_S_3_, meaning that the theoretical energy density is 760 Wh kg^−1^ [1]. It is important to note that the system presents certain security risks, and the elevated operation temperature increases the cost. In order to achieve a wider application of Na-S batteries, researchers have focused their efforts on room-temperature sodium–sulfur (RT Na-S) batteries. Similar to lithium–sulfur batteries, RT Na-S batteries typically use circular S_8_ molecules matched with Na metal, showing an excellent theoretical energy density, which can reach up to 1274 Wh kg^−1^ according to Equation (1) [2,3,4].

(1) $$S+2Na↔{Na}_{2}S$$

**Figure 1:**
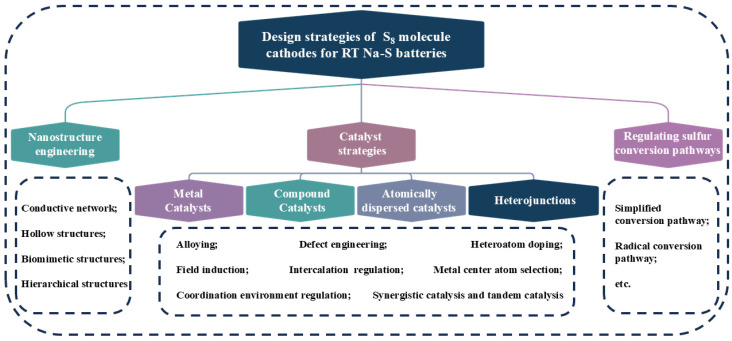
Design strategies of S_8_ molecule cathodes for RT Na-S batteries.

The conversion process of sulfur varies depending on the electrolyte used in the charge/discharge cycle. The charge/discharge process of RT Na-S batteries is complex, which means a series of polysulfide intermediates are generated during the reaction. Owing to the poor conductivity of the final discharge product (Na_2_S_2_/Na_2_S) and sulfur, the conversion reaction kinetics are sluggish, meaning that the electrode material cannot be fully converted and there is a low utilization of sulfur, which leads to a low coulombic efficiency, reduced discharge capacity, and poor rate performance [5,6]. In addition, the sluggish conversion reaction rate leads to the accumulation of sodium polysulfides (NaPSs), which are soluble, intensifying the “shuttle effect” and leading to the loss of active sulfur, which generates a gradual decline in the battery capacity and a serious self-discharge phenomenon [5,7]. Moreover, the huge volume strain of the electrode during charge and discharge cannot be ignored [7]. In addressing these primary issues, researchers have proposed numerous solutions. These include physical restriction [8] and introducing polar components with adsorptive and catalytic effects as catalysts [6] within the system. A comparative analysis reveals that the latter strategy is more effective in achieving the complete deep reduction of S_8_ molecules and is therefore highly favored. Herein, we present a summary and outline of the current research progress in terms of nanostructure engineering, catalyst strategies, and regulating sulfur species conversion pathways from the perspective of a high-performance host design strategy (Figure 1). An in-depth and thorough analysis of catalytic performance combined with the current research progress is presented from four perspectives: metal catalysts, compound catalysts, atomically dispersed catalysts, and heterojunctions, respectively. Finally, we analyze the bottlenecks and challenges encountered and provide some thoughts and suggestions on these problems.

## 2. Nanostructure Engineering

Nanostructured design has proven effective in lithium–sulfur batteries for enhancing electrochemical performance, so this strategy was extended to RT Na-S batteries. The reasonable design of host nanostructures is effective in improving battery performance. Reasonable nanostructure design should consider the following factors: 1. provide a large specific surface area for loading sulfur and curtailing catalyst size; 2. preset space to accommodate volume expansion during the charge–discharge process; and 3. provide a convenient electron/ion transfer pathway. Spheres [9,10], polyhedrons [11,12,13], sheets [14,15], hierarchical structures [16,17], and biomimetic structures [4,18] decorated with metal-based catalysts are designed as cathode hosts in RT Na-S batteries.

Various one-dimensional (1D) and two-dimensional (2D) materials have been introduced, such as carbon fibers [19,20,21], carbon nanotubes [13,22,23], rGO [24,25,26,27], Mxene [28,29,30], etc., to form multidimensional composite structures. These 1D or 2D carbon materials can effectively form a conductive network, which enhances electron conductivity. Additionally, the introduction of these 1D and 2D conductive networks enables the electrodes to achieve freestanding structures [19,31].

**Figure 2:**
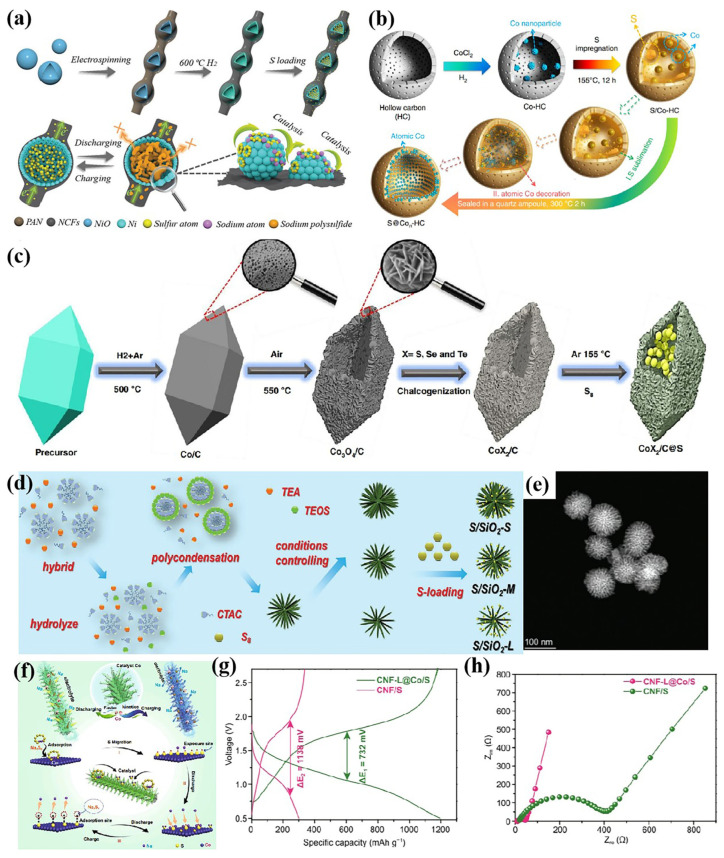
(**a**) Schematic illustration of S@Ni-NCFs [33]; (**b**) Schematic illustration of the synthesis of Co-HC [32]; (**c**) schematic illustration for the preparation the hollow polar bipyramid prisms [12]; (**d**) schematic diagram of the preparation of S/SiO_2_; (**e**) HRTEM images of the S/SiO_2_ [4]; (**f**) schematic of the preparation and the catalytic mechanism of CNF-L@Co/S; (**g**) comparison of GCD curves; and (**h**) comparison of EIS [18].

More complicated nanostructures have been designed for RT Na-S battery hosts. Some hollow structures have been designed, such as hollow spheres [10,32] and hollow polyhedrons [12], which are conducive to buffering the huge volume changes generated during the electrochemical redox process of S_8_ molecules, and provide a confinement effect on sulfur-containing species. Xu’s group designed a structure of hollow nickel units combined with a three-dimensional nitrogen-doped carbon fiber network (Ni-NCFs) to load S_8_ molecules (Figure 2a) [33]. The three-dimensional carbon fiber network facilitates rapid electron transport, and the hollow nickel spheres effectively limit the migration of sodium polysulfides and catalyze their reduction. The hollow structure can fully load sulfur, restrict the migration of sodium polysulfide, and buffer the volume strain during the discharge of the battery. Therefore, various hollow structures have been designed as hosts for RT Na-S batteries. Dou’s team designed hollow carbon spheres dotted with cobalt clusters to load sulfur molecules (Figure 2b) [32]. The battery has high sulfur utilization, as well as excellent cycling stability and rate performance. Xu’s group designed and prepared polar bipyramid prisms with CoX_2_ (X = S, Se, and Te) as hosts for RT Na-S batteries, which can not only efficiently load sulfur, but also adsorb polysulfides through polar chemical interactions and promote their catalytic conversion (Figure 2c) [12]. With this host, the battery can still work well even at a sulfur load of up to 9.1 mg/cm^2^, and exhibits a high capacity of 545 mAh g^−1^ at 0.5 C.

In order to achieve better performance, many bionic structures and hierarchical structures have been designed. On the one hand, this facilitates the transport of electrons and sodium ions, and on the other hand, more catalytic active sites are exposed, thereby significantly enhancing the conversion kinetics of sulfur-containing species. Some biomimetic structures have also been designed to host RT Na-S batteries. Xu’s group designed a Fe_2_N@NC with yolk–shell structure [34]. The S/YS-Fe_2_N@NC cathode exhibits up to a 1123 mAh g^−1^ specific capacity at 1 C, and a good rate performance (845 mAh g^−1^ at 2 C). Yang’s group designed a SiO_2_ host with an urchin-like structure, and the microstructure with abundant pore channels is conducive to loading sulfur, an adequate reaction, and rapid diffusion behaviors (Figure 2d,e) [4]. The S/SiO_2_ cathode exhibits outstanding electrochemical properties, and possesses a high reversible capacity up to 1370.6 mAh g^−1^ at 2 A g^−1^ and an impressive capacity retention of 97.5% at 5 A g^−1^ after 500 cycles. In addition, flower-like structures [14,15,35] and anthozoan-like structures [23] have also been designed for RT Na-S batteries.

A hierarchical structure is also one of the commonly employed strategies in nanostructure engineering. Xu’s group designed a 3D “branch-leaf” structure, where carbon fibers serve as branches and carbon sheets dotted with Co particles serve as leaves, thus fully exposing the catalytic site of Co (Figure 2f) [18]. Moreover, the structure has numerous electron/ion transfer pathways. So, a battery with 3D “branch–leaf” host exhibits a higher reversible capacity and lower overpotential and transfer resistance (Figure 2g,h).

In summary, the rational design of nanostructures can provide a larger specific surface area, catalytic sites, and more efficient charge transfer pathways, which are meaningful for improving RT Na-S batteries.

## 3. Catalyst Strategies

The introduction of a catalyst can restrict the migration of NaPSs through chemisorption. Heteroatom-doped carbon was initially used to immobilize polysulfide ions and catalyze the conversion of sulfur-containing species [36]. Subsequent studies have found that metal-based catalysts often have better performance [6]. The catalyst in RT Na-S batteries mainly promotes the transformation of long-chain NaPSs into insoluble Na_2_S_2_, and promotes the deep reduction of Na_2_S_2_ to the final product Na_2_S. Therefore, optimizing catalysts is essential to promote the transformation of sulfur-containing species and improve battery performance.

**Figure 3:**
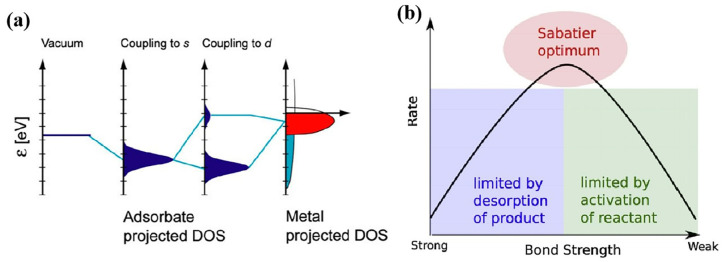
(**a**) Schematic of bond formation at a transition metal surface [37]; (**b**) schematic of Sabatier principle [39].

The energy band structure of the adsorbed substrates determines the adsorption strength according to d-band center models and p-band center models (Figure 3a) [37,38]. Surface adsorption and charge transfer determine the catalyst’s activity. According to the Sabatier principle, medium-strength surface adsorption is beneficial to obtaining higher catalytic activity (Figure 3b) [39]. For metal catalysts, the electronic structure of the d band, especially the location of the band center, determines the adsorption strength. Compared with metal catalysts, compound catalysts are relatively complex, due to the fact that the p-band electrons of non-metallic elements have a greater influence on the d-band electrons of metals through orbital hybridization. The most important means to regulate the intrinsic properties of catalysts is by regulating the energy band structure. Next, we will introduce the development and modification strategies of catalysts from the aspects of metal catalysts, compound catalysts, atomically dispersed catalysts, and heterojunctions.

### 3.1. Metal Catalysts

Metal catalysts are widely used in various types of catalysis. In RT Na-S batteries, metal catalysts often anchor NaPSs by forming M-S bonds, and subsequently transfer electrons to achieve NaPS conversion. Fe [40,41], Co [31,42,43,44], and Ni [33,45,46,47] are usually used to modify the conductive matrix to adsorb NaPSs and promote their rapid reduction to insoluble short-chain sodium sulfide, which can suppress the shuttle effect and enhance the utilization of sulfur. Co can effectively inhibit the dissolution and promote the conversion of NaPSs, and it interacts with NaPSs by forming a Co-S bond [23,32]. Xu’s team designed a series of Co-C composite hosts for an RT Na-S battery, all of which effectively improved battery performance [31,45,48]. Co can also promote the rapid reduction of NaPSs by forming a Co-S-Na molecular layer [18]. Moreover, Co can not only promote NaPS reduction, but also accurately regulate the 3D uniform nucleation mode of Na_2_S on the surface of the positive electrode material [42,44]. Wang’s group designed a Co-S-C@MC nanoreactor for S host, and the Co-S-C heterointerface can modulate the electronic structure and upshift the d-band center, thus possess high catalytic activity for NaPSs [49]. The battery achieved a 1310 mAh g^−1^ specific capacity, and a cycling stability over 2500 cycles. Ni is also an excellent catalyst for RT Na-S batteries [33,45]. It has been found that adjusting the Ni/Co ratio in a Ni-Co bimetallic catalyst enables multi-step controllable catalysis [46].

Au [50] and W [51] have also been used to catalyze the conversion of NaPSs, and have achieved excellent effects. As electrocatalysts, Au nanodots play a crucial role in converting Na_2_S_4_ to Na_2_S. Zhang’s group enabled the RT Na-S battery to achieve a high specific capacity and rate performance and excellent cycle stability by introducing a W nanoparticle electrocatalyst, which possess a 1160 mAh g^−1^ specific capacity, excellent rate performance, and cycle stability.

Metal particle catalysts exhibit outstanding performance in RT Na-S batteries. In pursuit of better intrinsic catalytic performance of metal catalysts, it is essential to adjust the electronic structure of the metal. Alloying is a common and effective method for the modification of metal electronic structure. Yu’s group designed and constructed an FeNi_3_@HC host, and the introduction of alloying elements increased the electron density of Ni, and enabled the conversion of NaPSs to be easier [52]. Furthermore, the catalyst carrier also has an important influence. Xie’s group designed electron-deficient centers in Co nanoparticles induced by the support; thus, the adsorption and catalytic conversion of polysulfide were enhanced [53].

### 3.2. Compound Catalysts

Compared with metals, compounds have a richer diversity, providing more selectivity for the selection of catalysts. The introduction of non-metallic elements can effectively adjust the d-band center of the catalyst, thereby enriching the means of regulating catalytic activity. Qian’s group studied the kinetics of polysulfide ion conversion catalyzed by cobalt-based compounds in Li-S batteries and found that the activity can be effectively regulated by adjusting the position of the p-band center [38].

Metal oxides are also widely used in RT Na-S batteries. Mn*_x_*O*_y_* [9,16,54,55], GeO*_x_* [56], CeO_2_ [57], TiO_2_ [58,59,60], V*_x_*O*_y_* [20,24], Nb_2_O_5_ [61], SiO_2_ [58,59,60], V*_x_*O*_y_* [20,24], Nb_2_O_5_ [61], SiO_2_ [4,62], and CoMoO_4_ [25] have been applied to RT Na-S batteries. The performance of metal oxides in RT Na-S batteries is not very prominent, probably because they are generally poor in conductivity and it is difficult to achieve electron transfer to NaPSs [63]. Nevertheless, due to the existence of special cases, some metal oxides also have outstanding performance in RT Na-S batteries, which will be discussed in detail later.

**Figure 4:**
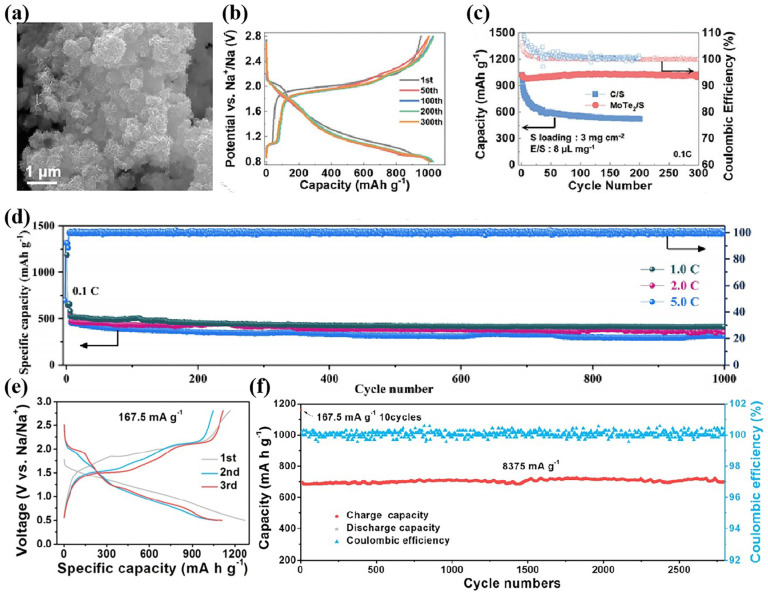
(**a**) SEM images of MoTe_2_; (**b**) GCD curve of MoTe_2_/S; (**c**) cycling performances of MoTe_2_/S [14]; (**d**) cycling performances of MoC@NHC [73]; and (**e**) GCD curve and (**f**) the cycling performance of Na-S@Fe_3_N-NMCN batteries [2].

NiS_2_ [64], CoS_2_ [65,66], CoSe_2_ [12,67], FeS_2_ [68,69], MnS [70], Ag_2_S [71], and molybdenum chalcogenides [14,15,26,35,72] have been investigated as catalysts in RT Na-S batteries. Among them, NiS_2_, CoS_2_, and CoSe_2_ possess good electrical conductivity, and are potential catalysts for RT Na-S batteries, exhibiting excellent performance in batteries. FeS_2_ possesses a low Na^+^ diffusion barrier, strong binding energy, and high affinity for polysulfides, and is an ideal sulfur host for immobilizing polysulfides and facilitating reversible conversion to Na_2_S [68]. As the catalyst of RT Na-S batteries, Ag_2_S releases Ag particles during the discharge process, which can significantly improve the battery performance [71]. Molybdenum chalcogenides have a two-dimensional layered structure, which allows for greater exposure of catalytic sites. MoTe_2_ with a 3D flower-like structure was used as the cathode host of RT Na-S batteries, and the battery showed a high reversible capacity and excellent cycle stability (Figure 4a–c) [14].

In recent years, metal nitrides and metal carbides have emerged as promising catalysts with good electrical conductivity. Mo*_x_*C [11,73], VC [21], Fe_3_C [74], Mxene [28,29], Fe*_x_*N [2,34], Mo*_x_*N*_y_* [75,76], Co*_x_*N [17], etc., were employed in RT Na-S batteries. At present, the RT Na-S batteries with these carbides as catalysts have shown excellent cycle stability. Mou et al. synthesized three-dimensional honeycomb hollow carbon spheres decorated by ultra-small α-MoC_1−*x*_ (MoC@NHC) as sulfur host materials for RT Na-S batteries [73]. The batteries exhibit excellent rate performance and cycle stability (Figure 4d). Metal nitrides have a low cost, excellent chemical stability, good electrical conductivity, and strong polysulfide anchoring capability. Li et al. grew Co_4_N sheets in situ on carbon cloth to form a 2D/3D hybrid structure (2D/3D Co_4_N-NC@CC), which was used as the host for RT Na-S batteries [17]. The RT Na-S cell with 2D/3D Co_4_N-NC@CC shows excellent long cycle stability (592 mA h g^−1^ at 1 C after 1000 cycles). Qiao’s group studied a series of molybdenum nitrides with different stoichiometric ratios for RT Na-S batteries, and Mo_6_N_5_ has a higher d-band center position than MoN and Mo_2_N, making it better able to catalyze NaPS conversion [75]. The material can achieve efficient Na_2_S electrodeposition; thus, the battery with Mo_6_N_5_ shows higher cycle stability. Qi et al. introduced Fe_3_N quantum dots into N-doped multilayered carbon networks (NMCNs) to form an Fe_3_N-NMCN host for RT Na-S batteries [2]. Fe_3_N has an excellent ability to inhibit NaPSs and promote NaPS conversion. The RT Na-S batteries with Fe_3_N-NMCNs show an excellent reversible capacity, rate, and cycle stability, and can achieve a specific capacity of 658.4 mA h g^−1^ at 10 C, with almost no capacity degradation after 2800 cycles at 5 C (Figure 4e,f).

As demonstrated above, compound catalysts exhibit outstanding performance in RT Na-S batteries. Common strategies for optimizing these catalysts include adsorption modulation, electronic state regulation, and intercalation modification.

#### 3.2.1. Adsorption Mode

**Figure 5:**
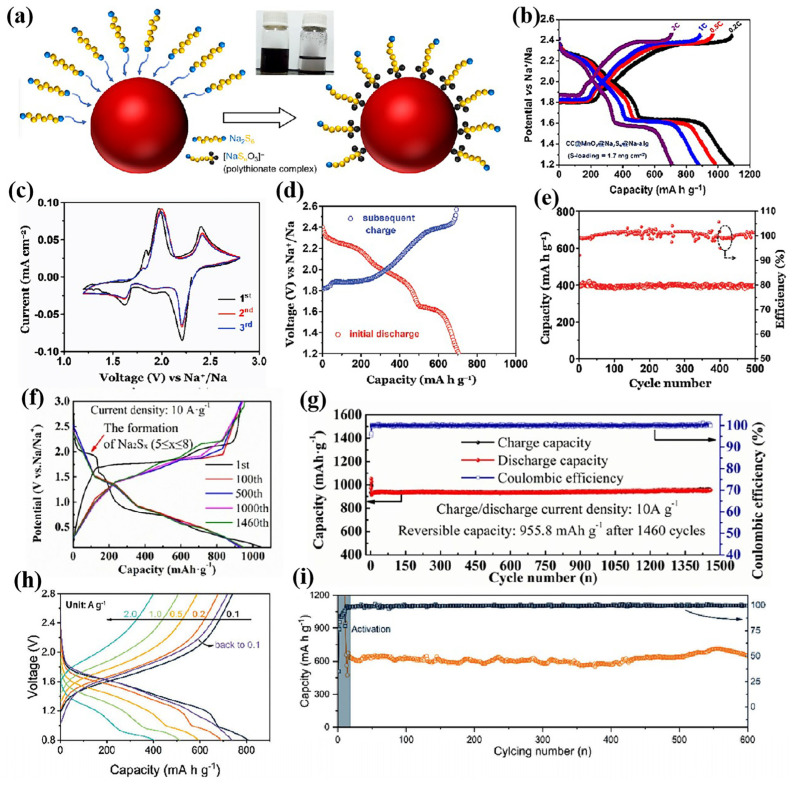
(**a**) Schematic diagram of Na_2_S_6_ adsorption on Mn*_x_*O*_y_* [54]; (**b**) GCD curve of the CC@MnO_2_@Na_2_S_6_@Na-alg cathode [16]; (**c**) CV curves and (**d**) GCD curve and (**e**) long-term cycling of S@CB@AlOOH cathode [77]; (**f**) GCD curve and (**g**) the cyclic performance of ASS [62]; and (**h**) GCD curve and (**i**) the cycling performance of S/Nb_2_O_5_-CNR [61].

Compared with metal catalysts, compound catalysts exhibit novel phenomena due to the presence of non-metallic elements. In metal catalysts, NaPSs are chemically adsorbed by the M-S bond between the metal and sodium polysulfide. However, due to the high valence of metals and the presence of non-metallic elements in compound catalysts, the bonding modes between sodium polysulfide and compound catalysts are more diverse. For example, NaPSs are oxidized to thiosulfate on the surface of Mn*_x_*O*_y_* and subsequently adsorbed on its surface (Figure 5a) [54]. Cathodes based on this chemisorption mode exhibit outstanding electrochemical properties. The rGO/S/Mn*_x_*O*_y_* cathode exhibits a high nominal voltage (1.81 V in discharge). And the CC@MnO_2_@Na-alg cathode shows remarkable cycling stability and rate performance (Figure 5b) [16].

Furthermore, NaPSs combine with compound catalysts through Na-O and Na-N bonds [2,62]. At present, these compound hosts that can bond with sodium ions have shown excellent performance. It should be pointed out that there is no d electron in amorphous silica (AS) and boehmite (AlOOH), and the d-band center theory may no longer be applicable in these cases. The chemisorption of NaPSs by amorphous silicon oxide and AlOOH is achieved by forming Na-O bond between NaPSs and the oxygen of these compounds. The S@CB@AlOOH cathode exhibits high reversibility and an excellent capacity retention ratio (Figure 5d,e) [77]. Similarly, an amorphous silica host also showed excellent performance. Surprisingly, the as-prepared sulfur electrodes of amorphous silica (ASS) exhibit a high reversible capacity and remarkable cycling stability, and maintain nearly 100% Coulomb efficiency and a 955.8 mAh g^−1^ capacity after 1460 cycles at 10 A g^−1^ (Figure 5f,g) [62]. In some compound catalysts, the catalyst binds to NaPSs by forming M-S bonds and Na-non-metallic bonds simultaneously. NaPSs are bound to orthorhombic Nb_2_O_5_ via Na-O and Nb-S bonds [61], and similarly FeN_3_ is bound to NaPSs by Fe-S bonds and Na-N bonds [2]; thus, they possess a strong affinity for polysulfides. Orthorhombic Nb_2_O_5_ is both sodiophilic and sulfurophilic, and promotes solid phase transformation of low-order sulfides through rapid Na ion diffusion. The S/Nb_2_O_5_-CNR cathode exhibits excellent rate and long cycle performance (Figure 5h,i) [61]. In summary, the adsorption mode is a critical factor influencing the performance of RT Na-S batteries. Therefore, modulating the adsorption mode represents an effective strategy for designing high-performance catalysts for RT Na-S batteries.

#### 3.2.2. Electronic State Regulation

For optimizing the performance of compound catalysts, many modification strategies have been explored, such as defect engineering, heteroatomic doping, and field induction. These methods have been proven effective in regulating electronic state, thereby enabling the development of high-performance catalysts.

**Figure 6:**
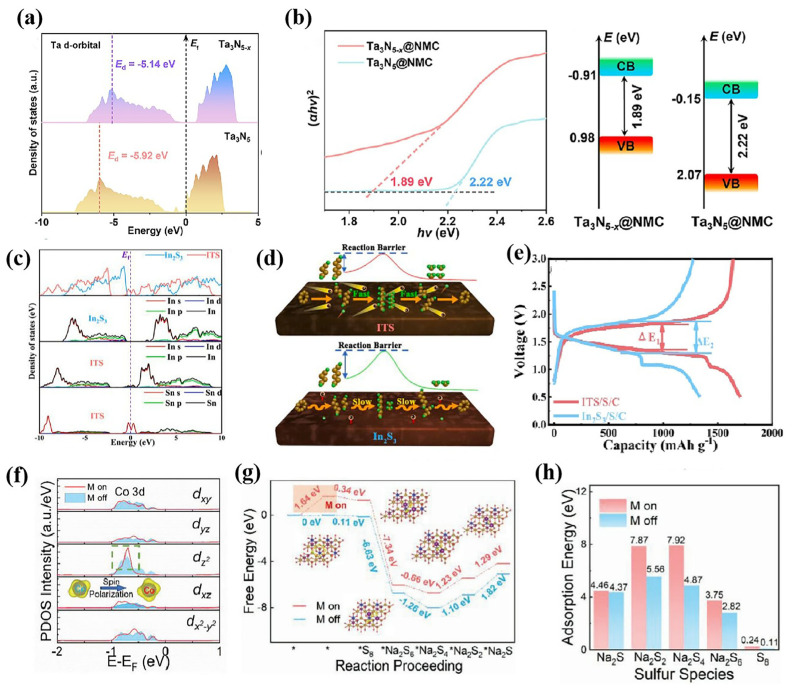
(**a**) DOS of Ta 5d orbital for Ta_3_N_5-*x*_ and Ta_3_N_5_; (**b**) Tauc plots and band diagrams of Ta_3_N_5_-x@NMC and Ta_3_N_5_@NMC [3]; (**c**) TDOS and PDOS of ITS and In_2_S_3_; (**d**) schematic illustration of the catalytic mechanism; (**e**) GCD curve of In_2_S_3_/S/C and ITS/S/C cathodes [78]; (**f**) PDOS of spin-polarized Co atom 3d orbital; (**g**) Gibbs free energy profiles and adsorption conformation of NaPS species on CoFe_2_O_4_; and (**h**) adsorption energy of CoFe_2_O_4_ surface for different sulfur species [81].

Vacancy defects are widely present in compounds, and the existence of vacancy defects can change the local electron density of compounds, significantly impacting the catalytic performance. Regulating vacancy defects has been extensively utilized to improve the catalytic activity of compounds. Chen’s group improved the adsorption and catalytic conversion of sodium polysulfide by inducing nitrogen vacancies in Ta_3_N_5_ (Figure 6a,b) [3]. According to DFT calculations, the generation of nitrogen vacancy reduces the band gap of Ta_3_N_5_, increases its electron conductance, shifts the d-band center of Ta_3_N_5_ upward, and strengthens the adsorption of NaPSs, thereby promoting the conversion from Na_2_S_4_ to Na_2_S. In addition, the researchers significantly improved the performance of the battery by constructing a rutile TiO_2_ and anatase TiO_2_ heterojunction, which generates a large number of oxygen vacancies at the interface to accelerate sulfur redox kinetics [59]. Vacancy defect induction is a general method to improve the catalytic performance of compounds, and has been applied in the modification of GeO_2_ [56] and CeO_2_ [57]. The doping of heteroatoms is also effective for improving the performance of compound catalysts. Doping Sn into In_2_S_3_ (ITS) regulates energy band structure, reduces its band gap, and improves its electrochemical performance (Figure 6c–e) [78]. Similarly, ITO (Sn doping In_2_O_3_) also showed excellent catalytic capacity for accelerating the reduction of Na_2_S_2_ to Na_2_S [79]. Moreover, heteroatom-doped Mxene also showed excellent performance, such as sulfur-doped Mxene and N-doped Mxene [28,29]. Vacancy defects and heteroatomic doping change the local electron state density, thereby improving its electrochemical performance. In addition, regulating the electron spin state of the catalysts can also improve the catalytic performance [80]. Researchers found that introducing a magnetic field could regulate the electron spin state of CoFe_2_O_4_, which enhanced the adsorption and catalytic conversion of NaPSs [81]. The magnetic field regulates the Co atom 3d orbital, which is beneficial to increasing the adsorption energy of NaPSs and reducing the kinetic barrier of NaPS transformation (Figure 6f–h).

#### 3.2.3. Intercalation Modification

**Figure 7:**
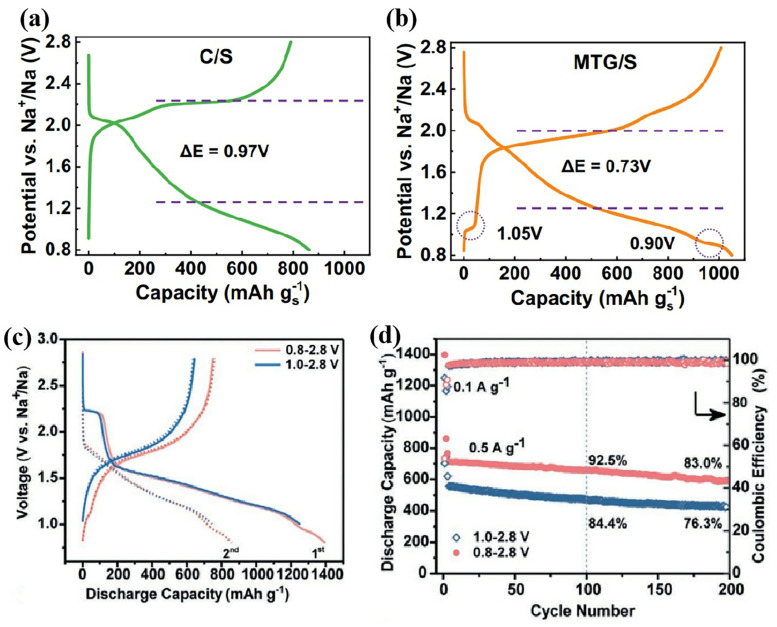
(**a**) The voltage profile of C/S cathode and (**b**) MTG/S cathode [88]; (**c**) GCD curves and (**d**) cycling performance of S/MoS_2_/NCS [87].

During the electrochemical reduction process, FeS_2_ [82] and MoS_2_ [83] may undergo changes in their properties due to the intercalation of metal ions, potentially resulting in unique characteristics. Intercalation modification is an effective strategy to improve the ability of hosts to promote polysulfide ion transformation. This strategy has been used in lithium–sulfur batteries, yielding significant improvements [84,85]. Within the charging–discharging voltage window, Na^+^ will intercalate into the catalyst in some cases, which improves the conductivity and catalytic activity of the catalyst. In RT Na-S batteries, FeS_2_ [86], MoS_2_ [87], and MoTe_2_ [88] catalysts have been found to be involved in local sodiation during the redox process, and their sodiation products can obtain stronger catalytic capacity for sulfur transformation (Figure 7). The ability of MoS_2_ to catalyze the NaPS conversion reaction is adjusted by adjusting the discharge depth, and the Na-S batteries based on this sodiated MoS_2_ host have outstanding performance, achieving a high capacity retention ratio of 84.4% at 1 Ag^−1^ after 2800 cycles [87].

### 3.3. Atomically Dispersed Catalysts

Atomically dispersed catalysts are efficient catalysts with a high utilization rate of catalyst atoms. The active units of atomically dispersed catalysts are composed of the central metal atom and the surrounding coordination atoms. The central metal atom is the actual catalytic active site, which means the selection of the appropriate central metal atom should not only consider the stability of the central metal atom, but also of factors such as the atomic size, orbital structure, electronic structure, and coordination environment. The d-electron state of the central metal atom is affected by the coordination environment.

**Figure 8:**
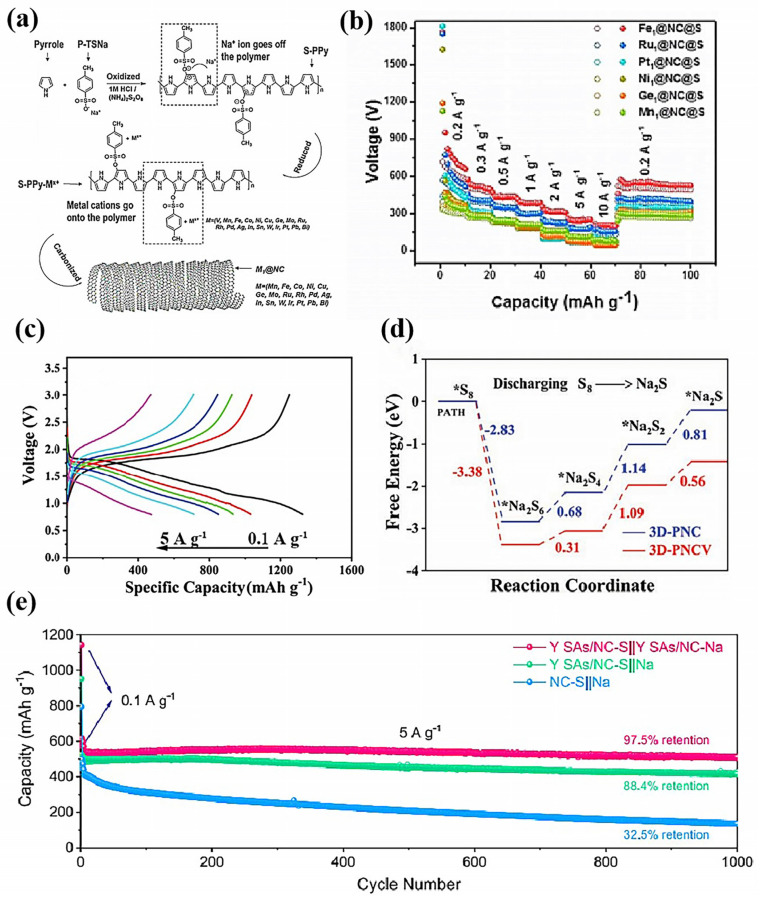
(**a**) The proposed synthetic approach and (**b**) rate performance of single-atom catalysts [89]; (**c**) GCD curves of the 3D-PNCV@S; (**d**) energy profiles for the polysulfide reduction of 3D-PNCV [91]; and (**e**) the cycling stability of the Y SAs/NC-S||Y SAs/NC-Na full cell [92].

At present, most of the atomically dispersed catalysts have d-orbital electrons in their center atoms, and their catalytic behavior is still consistent with the d-band center theory. The interaction between atomically dispersed catalysts and NaPSs is basically achieved through d-p hybridization between the central metal atom and sulfur element. Therefore, the choice of central metal atom plays a crucial role in determining the activity and selectivity of the catalyst. Chou’s group developed a universal method to synthesize a series of single-atom catalysts for emerging RT Na-S batteries, and investigated their electrocatalytic activity [89]. One of them, the Fe_1_ single atom, exhibited good catalytic performance (Figure 8a,b). Wang’s group compared and explored the electrochemical behavior of a series of single-atom catalysts with different centers of metal atom in RT Na-S batteries. They found that the Mn_1_ sites have a higher electron transfer capacity and higher selectivity for Na_2_S_2_ reduction in comparison to Fe_1_/Co_1_/Ni_1_/Cu_1_/Sn_1_ sites [90]. Yu’s group designed a single-atom catalyst of V (3D-PNCV) for RT-Na-S batteries, and the battery showed excellent cycle stability and rate performance, which benefits from the stronger adsorption of NaPSs and the lower NaPSs reduction Gibbs free energy on the 3D-PNCV substrate (Figure 8c,d) [91]. Li’s group designed a Y single-atom catalyst (Y SAs/NC) for an RT Na-S battery host, which is both sulfurophilic and sodiophilic, to be used in both the cathode and anode. And the Y SAs/NC-S||Y SAs/NC-Na full cell exhibited superdurable capacity retention (Figure 8e) [92].

Atomically dispersed catalysts have some unique functions due to their unique geometric structures and electronic states. Wu’s team designed a Mn single-atom catalyst that miraculously promotes the direct conversion of Na_2_S_4_ to Na_2_S [93]. Breaking with tradition, Wu’s team introduced the P-block metal element as the central atom of a single-atom catalyst (NHC-InN_5_) [94]. It was found by DFT calculations that the NHC-InN_5_ catalyst interacts with NaPSs through s-p hybridization, where the s orbitals of Na in NaPSs overlap with the p orbitals of NHC-InN_5_. And the degree of s-p orbital overlap has been identified as a key descriptor for catalytic sulfur species conversion reactions.

**Figure 9:**
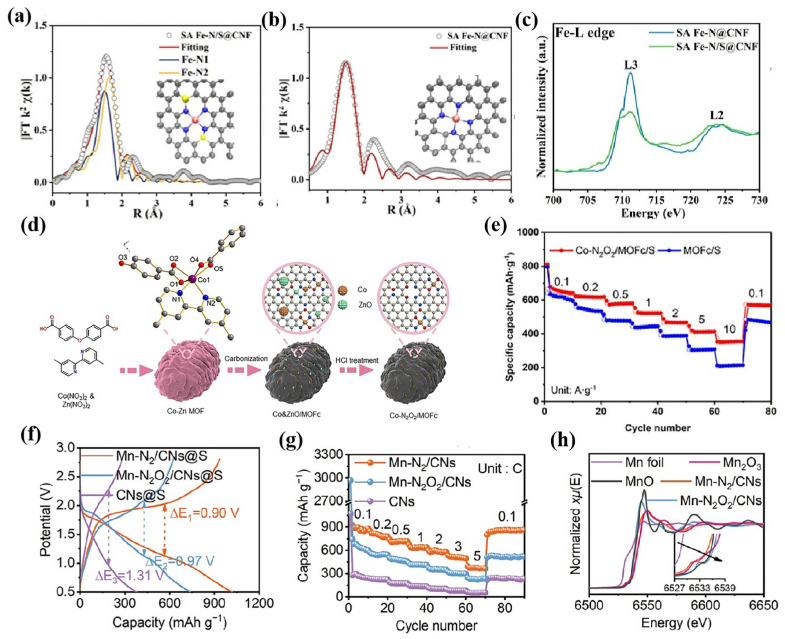
(**a**) Corresponding EXAFS fitting curves of SA Fe-N/S@CNF; (**b**) corresponding EXAFS fitting curves of SA Fe-N@CNF; (**c**) XANES spectra at the Fe-L edge of SA Fe-N/S@CNF and SA Fe-N@CNF [95]; (**d**) formation of Co-N_2_O_2_/MOFc composite; (**e**) rate performance of Co-N_2_O_2_/MOFc/S [96]; (**f**) GCD curves of Mn-N_2_/CNs@S; (**g**) rate performances of Mn-N_2_/CNs@S; and (**h**) Mn K-edge XANES spectra [98].

Coordination atoms are also crucial for stabilizing the central metal atoms and improving their catalytic properties. Yu’s group introduced two second-shell S atoms connecting to the N atoms of Fe-N bonds to form the coordination structure of Fe-N_4_S_2_ (Figure 9a) [95]. The second shell sulfur atom can cause the d-band center of monatomic iron to shift upward, resulting in stronger catalytic activity than the traditional Fe-N_4_ site in the sulfur conversion reaction, and the obtained S cathode delivers a high initial reversible capacity of 1590 mAh g^−1^. Gao’s group designed and prepared a cobalt monatomic catalyst with N/O double coordination (expressed as Co-N_2_O_2_/MOFc) (Figure 9d) [96]. Compared with Co-N_4_, the d-electron density of the Co atom can be further regulated by introducing an oxygen atom. Thus, the d-p hybrid of Na_2_S*_x_* adsorbed on Co-N_2_O_2_/MOFc was enhanced, the adsorption energy of Na_2_S*_x_* was increased, the Na_2_S decomposition energy barrier was decreased, and the kinetics of the NaPS conversion reaction was promoted [96]. Similarly, Cu single-atom catalysts with a Cu-N_2_O_2_ coordination structure also showed an excellent ability to promote NaPS conversion [97].

Regulating the coordination number of the central atom can effectively modify the properties of single-atom catalysts [99,100]. Compared with Zn-N_4_, unsaturated Zn-N_2_ has an asymmetric electron distribution, which is conducive to anchoring and activating NaPSs, accelerating the NaPS conversion process and reducing the Na_2_S decomposition energy barrier [99]. Novel asymmetric Mn-N_2_ structures were implanted into nitrogen-doped carbon nanofibers (Mn-N_2_/CNs) by thermal NH_3_ etching of symmetric Mn-N_2_O_2_ structures [98]. The Mn-N_2_ structure promotes the binding affinity and catalytic NaPS conversion on account of the strengthening of the d-p orbital hybridization between the Mn and S of NaPSs. Compared with Mn-N_2_O_2_, the cathode based on Mn-N_2_ exhibits a lower overpotential and better rate performance (Figure 9f,g). Mn-N_2_/CNs@S achieves a high capacity of 458 mAh g^−1^ at 3 C and a capacity decay rate of only 0.23% in 2300 cycles [98]. Unsaturated coordination can effectively regulate the properties of atomically dispersed catalysts. Likewise, supersaturated coordination can also affect catalytic activity. Compared to In-N_4_, In-N_5_ single-atom catalysts exhibited a lower energy barrier in the sulfur species conversion process [94].

In addition to regulating the coordination atom type and coordination number of the central atom, regulating the interaction between metal atoms can also well regulate the performance of atomically dispersed catalysts. Cui et al. substantiated through theoretical calculations that the d-d orbital coupling between the central metal atoms significantly affects the catalytic performance of sulfur species transformation in atomically dispersed catalysts, which offers a new perspective for the design and screening of dual-atom catalysts [101]. Moreover, a linearly interlinked Fe-N*_x_*-Fe catalyst was designed, and the study found unique metal–iron bonds that contribute to the transfer of electrons to the sulfur cathode, thus accelerating the reaction kinetics in the NaPS conversion process [102]. The development of dual-atom catalysts represents one of the potential future directions in the evolution of atomically dispersed catalysts.

In addition to forming atomically dispersed catalysts based on M-N coordination on carbon materials doped with heteroatoms, such as O and N, atomically dispersed catalysts can also be formed on inorganic compounds. Atomically dispersed monolayer MoS_2_ clusters and dual active sites of Mo single atom were loaded onto sulfur-doped graphene frames to achieve an 80.9% sulfur-loading cathode material (S@MoS_2_-Mo_1_/SGF) and obtain excellent electrochemical performance. Under the current density of 0.1 A g^−1^, the reversible capacity of the first cycle of the S@MoS_2_-Mo_1_/SGF cathode material reaches 1017 mAh g^−1^, and the capacity decay rate is only 0.05% per cycle. The dual-active-site MoS_2_-Mo_1_ can form a delocalized electron system, which can not only improve the reactivity of sulfur, but also promote the reversibility of the reaction between sodium and sulfur, thus inhibiting the shuttle effect. Zhou et al. prepared a highly dispersed Mo monoatom catalyst on the MoS_2_ substrate through electrochemical reconstruction, which enhanced the overlap between the d orbital of Mo and the p orbital of s in Na_2_S_6_, promoted the strong adsorption of NaPSs, and thus had a significant impact on the electrochemical performance [103].

### 3.4. Heterojunctions

Catalysts are often selective, and it is difficult for a single catalyst to meet the demands of the complex multi-step reaction system of RT Na-S batteries. Developing multifunctional catalysts is of great significance for improving RT Na-S batteries. Heterojunction catalysts have been widely studied, and it is significant to construct heterojunctions to enhance the catalytic activity of the sulfur-containing species conversion reaction [104,105,106]. On the one hand, the construction of heterogeneous structures has a significant effect on regulating the electronic state of the catalyst. An internal electric field is formed at the heterojunction interface, reorganizing the electron distribution of the electrocatalyst, accelerating the charge transfer, and increasing the active sites, thus enhancing the activity of the electrode reaction. On the other hand, the composite components in the heterojunction can realize multi-function synergistic catalysis and tandem catalysis.

**Figure 10:**
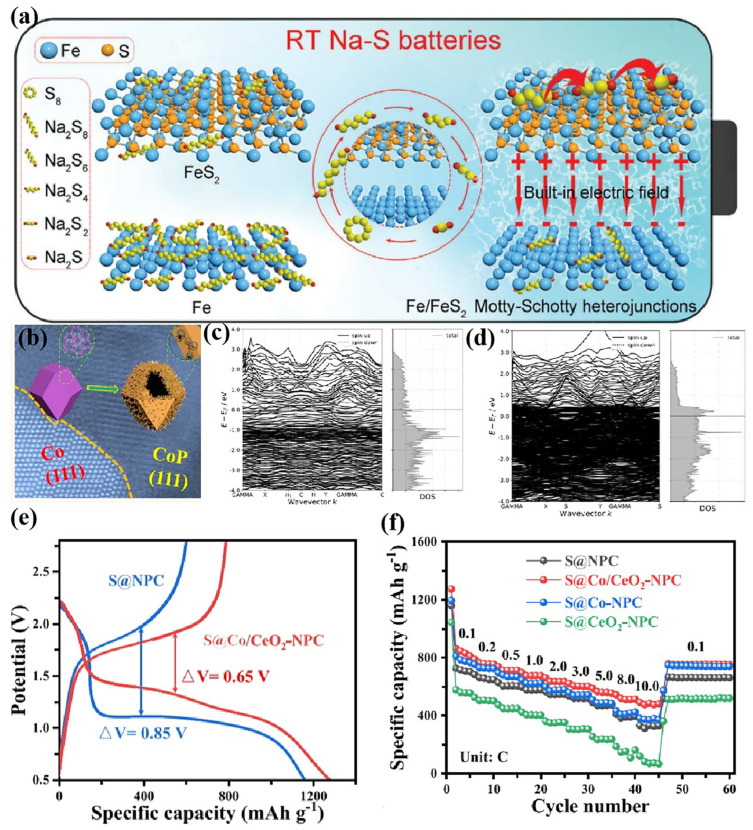
(**a**) Schematic illustration of Fe/FeS_2_ heterojunction-enhanced NaPS conversion [107]; (**b**) HRTEM and schematic illustration of CoP-Co heterojunction; (**c**) band structure and DOS of the CoP surfaces; (**d**) band structure and DOS of the CoP-Co surfaces [108]; and (**e**) GCD curves and (**f**) rate of S@Co/CeO_2_-NPC [13].

Metal-compound-type heterojunctions are often used to catalyze conversion reactions of sulfur species, exhibiting excellent ability to inhibit the shuttle effect and promote NaPS conversion. In Fe-FeS_2_ heterojunctions, a built-in electric field is formed at the contact interface between Fe and FeS_2_ (Figure 10a) [107]. The adsorption capacity of iron for NaPSs and the catalytic effect of FeS_2_ on NaPSs were enhanced greatly, improving performance when combined with the rapid transfer effect of the built-in electric field. In Co-CoP heterojunctions, the electron distribution is reorganized, and the Co-CoP has a larger bandwidth and a higher Fermi level, which may bring substantial benefits in terms of adsorption properties and charge transfer (Figure 10b–d) [108]. Co-CeO_2_ integrates the strong adsorption capacity of CeO_2_ for NaPSs and the property of Co promoting the rapid transformation of NaPSs, reducing the overpotential and enhancing the rate performance of batteries (Figure 10e,f) [13]. Moreover, Ni-MnO_2_ [10], Ni-Ni_3_N [109], Co-Co_3_C [110], and Co-MG [30] heterojunction catalysts have also been developed, which significantly improve the electrochemical performance of RT Na-S batteries.

**Figure 11:**
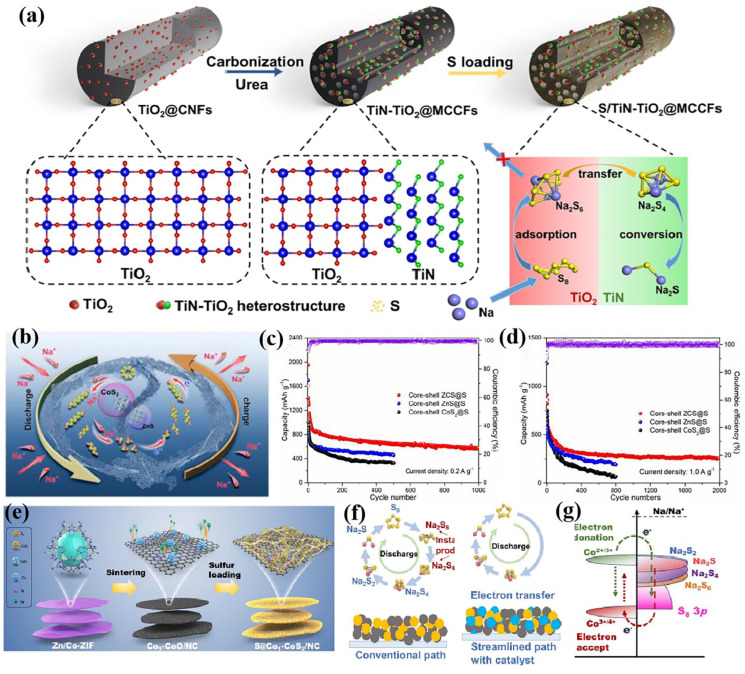
(**a**) Schematic of the design principle of the TiN-TiO_2_ heterostructure [111]; (**b**) schematic illustration of synergistic electrocatalytic in CoS_2_-ZnS core–shell; (**c**,**d**) cycling performances of core–shell ZCS@S (CoS_2_-ZnS@S) at different rate [119]; and (**e**) schematic illustration of S@Co_1_-CoS_2_/NC synthetic process and (**f**) redox path and (**g**) electron transfer [120].

Compound-based heterojunctions are similar to metal compound heterojunctions in that they also have built-in electric fields and electron rearrangements, but are more complex. The heterogeneous interfaces formed by the two compounds not only inherit their respective functions, but also produce synergistic properties that exceed the individual benefits they provide. On one hand, compound-based heterojunctions also improve their performance by integrating the strong adsorption and fast conversion of NaPSs, and the abundant heterointerfaces facilitate electron/ion transport and accelerate NaPS conversion. TiO_2_ shows a strong adsorption capacity for different sulfur species. However, the performance of TiO_2_ catalytic NaPS reduction is not outstanding due to poor electrical conductivity [58,111]. Ye et al. designed a TiN-TiO_2_ heterojunction combining a TiN accelerator with a TiO_2_ inhibitor [111]. The polysulfide is strongly anchored by TiO_2_ and rapidly migrates from the TiN-TiO_2_ interface to the TiN surface for electrocatalytic deposition (Figure 11a). The RT Na-S battery with a TiN-TiO_2_ heterojunction shows excellent performance. Yu’s group designed a S/CoS_2_-CoSe_2_@CNFs cathode for RT Na-S batteries, and integrated the capacity of CoS_2_ for trapping NaPSs and the catalytic capacity of CoSe_2_ for NaPS conversion [112]. Other heterojunction combinations exhibit similar effects, such as Ti_3_C_2_T_x_-Ni(OH)_2_ [113], MoC-W_2_C [114], Mo_2_N-W_2_N [105], MoS_2_-MoN [115], TiO_2_-Ti_3_C_2_, TiO_2_-BaTiO_3_ [104], and Co_1_-ZnS [116]. Xu et al. introduced amorphous NiB as a catalyst for RT Na-S batteries [117]. Interestingly, during the charging and discharging process of batteries, amorphous Ni-B tends to be converted into NiS*_x_*, forming a NiB-NiS*_x_* interface, which shows higher catalytic performance and promotes the kinetics of sulfur species transformation at high current densities and in low-temperature environments.

On the other hand, the coordination of the components in the heterojunction can realize the relay catalysis of the NaPS multi-step conversion process, which is conducive to the continuous and rapid conversion of sulfur species. This type of tandem catalysis is significant for achieving a high sulfur utilization rate and the reversibility of sulfur species conversion. Yang’s group designed a ZnS-NC@Ni-N_4_/S cathode, where ZnS promotes the conversion of S_8_ to Na_2_S*_x_* (4 < x ≤ 8), and Ni-N_4_ promotes the deep reduction of NaPSs to Na_2_S [118]. Moreover, a CoS_2_-ZnS core–shell structure was designed to inhibit the shuttle effect and accelerate the conversion process of sulfur-containing species (Figure 11b) [119]. CoS_2_ catalyzes the reduction of NaPSs, and ZnS can promote the oxidation of Na_2_S to sulfur, which is beneficial to improving the reversibility of sulfur species conversion.

Miraculously, the introduction of heterojunction catalysts can also regulate the reaction pathways of sulfur-containing species. Wang et al. designed and prepared a S@Co_1_-CoS_2_/NC cathode where CoS_2_ particles serve as an electron reservoir, and Co_1_ and the electron reservoir work collaboratively to enhance simplified redox pathways and directly reduce sulfur molecules to Na_2_S_4_ (Figure 11f) [120]. These “Streamline Sulfur Redox Reactions” are crucial for improving battery performance.

## 4. Regulating Sulfur Species Conversion Pathways

Catalysts offer a significant advantage by providing abundant active sites, which facilitate accelerated electron transport, inhibiting the aggregation of intermediates and enhancing the conversion reactions among sulfur species [6]. Therefore, the introduction of a catalyst into the S host matrix can effectively promote the transformation of NaPSs during the discharge process and reduce the oxidation energy barrier of Na_2_S during the charge process. These improvements can significantly enhance the redox kinetics and cycle stability of RT Na-S batteries. However, the redox pathway of sulfur species in RT Na-S batteries is still not very clear. The performance of RT Na-S batteries was improved with half the effort by adjusting the species of intermediate sulfur and developing a new REDOX mechanism for NaPSs. The general reaction path of sulfur species is that the S_8_ molecule is first transformed into Na_2_S*_x_* (6 ≤ x ≤ 8), then reduced to Na_2_S_4_, then further transformed to Na_2_S_2_, and finally reduced to Na_2_S [121,122]. With the continuous deepening of research, some new conversion pathways have been discovered, which is very meaningful to improve battery performance. As mentioned earlier, Wu’s team found that the manganese monoatomic catalyst could promote the direct reduction of sodium polysulfide to Na_2_S without the conversion of Na_2_S_4_ to Na_2_S_2_ [87]. And the S@Co_1_-CoS_2_/NC cathode designed by Wang et al. enables the direct conversion of S_8_ to Na_2_S_4_ without the formation of the intermediate Na_2_S*_x_* (6 ≤ x ≤ 8) [93,120]. More and more methods are being tried to regulate the conversion pathway of sulfur species.

**Figure 12:**
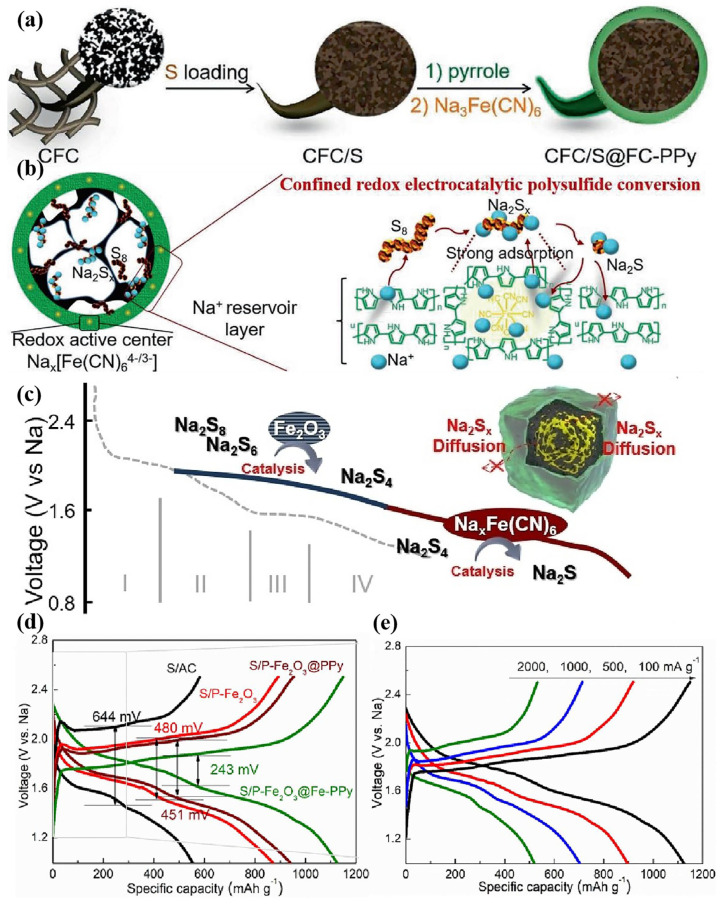
(**a**) Scheme diagram of CFC/S@FC-PPy preparation process; (**b**) scheme diagram of polysulfide conversion of CFC/S@FC-PPy [123]; (**c**) schematic diagram of the polysulfide conversion processes; and (**d**,**e**) comparison of S/P-Fe_2_O_3_@Fe-PPy GCD curves [124].

As shown in Figure 12a,b, Lu’s team found that Fe (CN)_6_^4−^ free radicals exhibited excellent electrocatalytic properties in RT Na-S batteries [123,124]. NaxFe (CN)_6_^4−/3−^ can act as a redox mediator in the process of catalyzing NaPS conversion, due to the change in the valence state of the iron element, which can achieve a direct conversion of Na_2_S_4_ to Na_2_S without undergoing the formation of a solid Na_2_S_2_ incomplete product. The redox mediator can also reduce the oxidation energy barrier of Na_2_S and improve the reversibility. To further improve the electrochemical performance, a bidirectional tandem catalyst was designed by replacing porous carbon fiber with porous Fe_2_O_3_. Fe_2_O_3_ promoted the formation of Na_2_S_4_, while the redox mediator promoted deep reduction to form Na_2_S, enabling continuous sulfur conversion [124]. And the battery shows a low polarization voltage and high rate performance (Figure 12c–e).

**Figure 13:**
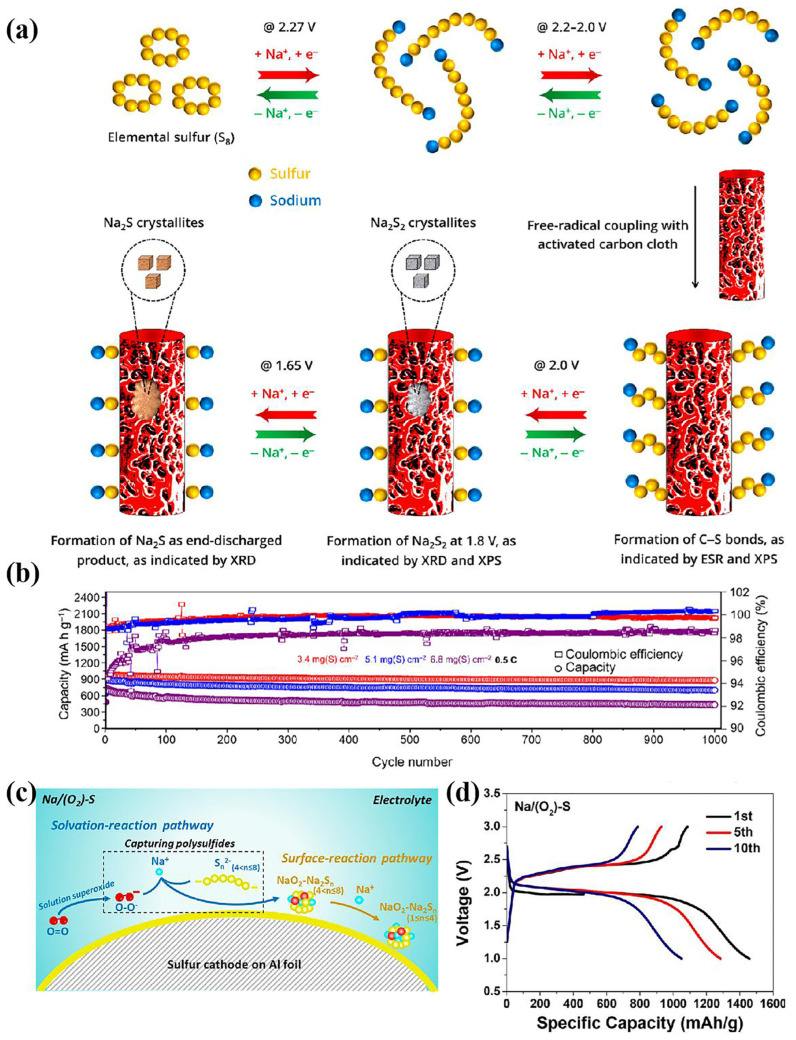
(**a**) Reaction mechanism describing Na_2_S_6_@ACC [127]; (**b**) the cycle performance of Na_2_S_6_ @ITO@ACC [79]; (**c**) schematic illustration of the discharge reaction pathway of Na/(O_2_)-S batteries; and (**d**) the GCD curves of the Na/(O_2_)-S cell [128].

In Li-S batteries, sulfur radicals can accelerate the conversion of polysulfide. Triggering and stabilizing sulfur radicals is an important approach to improve the kinetics of sulfur conversion [125,126]. The presence of tri-sulfur free radicals has been demonstrated in RT Na-S batteries. Mitra’s group confirmed the formation of the radical monoanions S_3_^−^ during discharge by in situ Raman spectroscopy. Na_2_S_6_ is dissociated by a homolysis bond to produce relatively stable S_3_^·−^ free radical monoanions, and the C-S bond forms a suspension bond on the matrix surface by coupling with polysulfide ions (Figure 13a) [79,127]. RT Na-S cells based on this new redox pathway (Equation (2)) can achieve a high nominal battery potential of 1.85 V.

(2) $${Na}_{2}S_{6}↔{2{NaS}_{3}}^{\cdot }+4{Na}^{+}+4e^{-}\to 3{Na}_{2}S_{2}$$

Based on S_3_^−^ free radical conversion pathways, combined with the advantages of ITO promoting the deep reduction of Na_2_S_2_, the battery exhibits a reversible capacity of up to 1167 mAh g^−1^ at 0.2 C and low capacity decay rate (Figure 13b) [79]. And the battery still performs well at −10 °C.

In addition to regulating the formation of sulfur-containing species in the redox process. Xu’s group doped the S element with oxygen which is introduced into the electrolyte to form nanoscale NaO_2_-Na_2_Sn (1 < n ≤ 4), thus changing the reaction path of the positive sulfur electrode (Figure 13c) [128]. These intermediates act as fixed polysulfides, achieving a high capacity and low overpotential. The nanoscale NaO_2_-Na_2_Sn particles are insoluble, which inhibits the shuttle effect of polysulfides, resulting in low self-discharge and excellent cycling performance. Analogously, the introduction of a small amount of selenium also played a good role in promoting the conversion of sulfur. Sulfur was loaded onto ZnSe/HSC and treated at high temperature to form S@Se-ZnS/HSC. The in situ introduction of selenium into sulfur, which enhances conductivity and forms S-Se bonds, thereby inhibited the shuttle phenomenon. And the battery still has an excellent capacity retention ratio at −10 °C [129].

## 5. Conclusions and Outlook

In the development of RT Na-S batteries, numerous solutions have been proposed to address the challenges of the poor conductivity of sulfur cathodes, serious volume expansion, and the shuttle effect of intermediate products. Strategies such as nanostructure engineering, catalyst design, and the regulation of sulfur species conversion pathways have been explored. Conductive networks have been introduced, and hollow structures, biomimetic structures, and hierarchical architectures have been designed to serve as hosts, achieving notable success. Strategies such as alloying, defect engineering, field induction, and coordination environment modulation, and so on have been applied to the design and optimization of metal catalysts, compound catalysts, atomically dispersed catalysts, and heterojunction catalysts. These approaches have significantly enhanced the intrinsic catalytic performance of the catalysts, facilitating the conversion of polysulfide molecules. Simplifying the polysulfide conversion process, exploring radical-mediated polysulfide conversion pathways, and leveraging heteroatom-assisted polysulfide conversion have emerged as promising methods for regulating polysulfide conversion pathways in RT Na-S batteries, making substantial contributions to improving their performance. These strategies have effectively enhanced the performance of RT Na-S batteries. Herein, we summarize the electrochemical performance of RT Na-S batteries, as shown in Table 1.

**Table 1:** A summary of the electrochemical performances of RT Na–S batteries.

Host	Sulfur Loading Mass (mg cm^−2^)	Current Density	Cycle Number	Specifc Capacity (mAh g^−1^)	Ref.
Bipyramid prisms CoS_2_/C	4.4	0.5 C	800	675	[12]
Ultrafine α-MoC_1-*x*_	2	1 C 5 C	1000 1000	418.4 312.6	[73]
Urchin-Like SiO_2_	0.7–1.1	2 A g^−1^ 5.0 A g^−1^	1050 500	1280.8 1132.6	[4]
2D/3D Co_4_N–NC@CC	1	1 C	1000	592	[17]
Amorphous Silica	0.7–1.1	10 A g^−1^	1460	955.8	[62]
Fe_3_N/C	~2.8	8375 mA g^−1^	2800	696	[2]
Ta_3_N_5-*x*_@NMC	~1	3.35 A g^−1^	2000	730	[3]
TiO_2_ anatase/rutile (Q_V_TRA)	-	1 C	1000	759	[59]
CNF/CoFe_2_O_4_	0.5–0.6	1 C	2700	542	[81]
Sodiated MoS_2_	1.5	0.2 A g^−1^ 1 A g^−1^	800 2800	774.2 360.7	[87]
3D-PNCV	-	5 A g^−1^	800	445	[91]
Y SAs/NC	-	5 A g^−1^	1000	510	[92]
Mn-N_2_/CNs	~0.9	3 C	2300	458	[98]
Fe-N*_x_*-Fe	1.5–2	10 A g^−1^	5000	325	[102]
Co-CeO_2_	1.5	5 C	1000	561.1	[13]
ZnS-NC@Ni-N_4_	1.4	5 A g^−1^	2000	650	[118]
MoC-W_2_C-MCNFs	2	0.2 A g^−1^ 4 A g^−1^	500 3500	640 200	[114]
BaTiO_3_-C-TiO_2_	1.2–1.4	1 A g^−1^ 2 A g^−1^	1400 3000	524.8 382	[104]
ITO@ACC	6.8	0.5 C	1000	445	[79]

However, several scientific questions remain to be resolved: (i) the sulfur loading of RT Na-S batteries is generally low, making it difficult to meet the requirements for commercial applications; (ii) the conversion mechanisms and pathways of NaPSs remain unclear; (iii) research on metal-based catalysts has primarily focused on d-block metal elements, with limited exploration of other metal-based systems; and (iv) single-function catalysts are insufficient to address the complex multi-step reaction system of RT Na-S batteries. In subsequent research, the following aspects need significant attention: (i) the large-scale production of cathode materials: at present, RT Na-S battery technology remains in the research and development phase, necessitating further advancement and industrialization to meet the demands of practical applications; (ii) clarifying the sulfur conversion mechanism in different electrolyte systems; (iii) identifying the transient transformation process of NaPSs and the influencing factors of the transformation pathway of sulfur-containing species by characterization techniques in situ; (iv) utilizing AI technology to identify the descriptors for catalyst interactions with NaPSs; (v) expanding the selection range of catalysts, such as p-block metal-based catalysts; and (vi) developing multifunctional catalysts with synergistic capabilities, such as multi-atom catalysts. In addition to improving the cathode, the optimization of electrolyte systems such as high-concentration electrolytes, local high-concentration electrolytes, and the development of solid and quasi-solid electrolytes with high sodium ion conductivity are also crucial to achieve safe and efficient applications of RT Na-S batteries.

## Data Availability

No new data were created or analyzed in this study.
